# Cardiac testing choices by physician specialty in the CMR-IMPACT trial

**DOI:** 10.1016/j.ajem.2025.01.073

**Published:** 2025-01-27

**Authors:** Michael W. Supples, Anna C. Snavely, Nicklaus P. Ashburn, Lauren E. Koehler, Jason P. Stopyra, Carolyn J. Park, Sujethra Vasu, Michael Kutcher, Gregory Hundley, Simon A. Mahler, Chadwick Miller, CMR-IMPACT Research Group

**Affiliations:** a Department of Emergency Medicine, Wake Forest School of Medicine, Winston-Salem, NC, USA; b Department of Biostatistics and Data Science, Wake Forest School of Medicine, Winston-Salem, NC, USA; c Section on Cardiovascular Medicine, Department of Internal Medicine, Wake Forest School of Medicine, Winston-Salem, NC, United States of America; d Division of Cardiology, Department of Internal Medicine, Virginia Commonwealth University, Richmond, VA, USA; e Department of Epidemiology and Prevention, Wake Forest School of Medicine, Winston-Salem, NC, USA; f Department of Implementation Science, Wake Forest School of Medicine, Winston-Salem, NC, USA

**Keywords:** Chest pain, Acute coronary syndrome, Angiography, Cardiac magnetic resonance

## Abstract

**Background:**

Heterogeneity is observed in the care of patients with chest pain. We investigate the association of physician specialty and diagnostic testing among patients admitted for suspected acute coronary syndrome (ACS).

**Methods:**

This is a secondary analysis of the CMR-IMPACT multicenter randomized controlled trial in which patients with suspected ACS were admitted and randomized to undergo invasive angiography or non-invasive CMR stress imaging. Admitting physician was dichotomized to interventional cardiologist (IC) or not (e.g. hospitalist). We describe adherence to protocol and angiography during the index visit by treatment arm and admitting physician specialty. A generalized estimating equation accounting for clustering within physician was used to evaluate significance and adjusted for randomization arm.

**Results:**

The 258 enrolled patients from 2013 to 2018 had a mean age of 60.7 (SD ± 10.9) years, 40.3 % (104/258 were female), and 64.7 % (167/258) were white race. ICs were the admitting physicians for 50.4 % (130/258) of the patients. Index angiography was performed more often among patients admitted by IC versus other physicians, 65.4 % (85/130) versus 53.1 % (68/128), respectively; aOR 1.75 (95 % CI 1.14–2.68). Among patients randomized to an invasive strategy, higher protocol adherence was observed in those admitted by IC [85.3 % (58/68)] versus other physicians [64.5 % (40/62)]; OR 2.82 (95 % CI 1.08–7.38). For patients randomized to the CMR-based strategy, adherence to protocol was similar for IC [67.7 % (42/62)] and other physicians [66.7 % (44/66)]; OR 0.82 (95 % CI 0.35–1.94).

**Conclusion:**

Invasive angiography was more frequent among patients admitted by interventional cardiologists compared to other physicians.

## 1. Introduction

Heterogeneity is observed in the care of patients with chest pain [1–6]. Physician education and specialty are associated with variations in practice patterns among several diseases, including acute coronary syndrome (ACS) [2–4,7]. Several factors may drive this association. Reimbursement potential has been observed as a factor in diagnostic and treatment choices among various medical specialties. Variations have also been observed in cardiac stress tests ordered depending on the ordering clinician’s ability to bill technical or professional fees [8–10]. Two previously published studies found little to no significant association of physician specialty with the cardiac testing choices [5,6]. Given the limited published evidence, the association of physician specialty on the choice of diagnostic modality used to evaluate patients for ACS remains uncertain.

Invasive coronary angiography is a common strategy used to evaluate patients for ACS and is associated with a risk of complications due to its invasive nature. Cardiac magnetic resonance (CMR) imaging is a non-invasive approach to investigate chest pain, particularly in more complex patients such as those with detectible to mildly elevated serum troponin results. A CMR-based strategy reduces the rate of invasive coronary angiography [11]. In the CMR-IMPACT randomized controlled trial, significant heterogeneity was observed in diagnostic testing, with one third of patients allocated to CMR receiving another form of cardiac testing, and similar cross over was also observed in the invasive angiography arm [11]. It is unclear whether physician specialty or training impacted the choice of cardiac testing modality.

We hypothesize that physician specialty is associated with variations in cardiac testing modalities. In this secondary analysis, we examine whether the frequency of invasive coronary angiography differs by the admitting physician specialty (interventional cardiologist versus other physicians).

## 2. Methods

### 2.1. Study design, setting and participants

This is a secondary analysis of the CMR-IMPACT trial, a randomized, controlled, multicenter clinical trial, which enrolled patients with detectable to mildly elevated troponin measures across 3 US emergency department sites from September 2013 to July 2018 Only patients enrolled at Wake Forest Baptist Medical Center were included in this analysis due to availability of admitting physician specialty data. All participants provided written informed consent for study participation before being randomized to an invasive-based or CMR-based strategy. This study was approved by the institutional review boards of the participating sites.

Methods of the CMR-IMPACT trial have been previously described [11]. Patients at least 21 years old presenting with symptoms concerning for ACS who had at least one serum troponin value between the limit of detection and 1.0 ng/mL were screened. Institutional practice at the time of the study was to admit all patients being investigated for ACS who had an elevated troponin, regardless of risk score. Consistent with this practice, all participants in the trial were placed on an inpatient service for completion of their evaluation. Enrolled participants were randomized to one of the two treatment arms with equal probability using random permuted block randomization.

### 2.2. Study procedures and data collection

Participants were admitted preferentially to the cardiology service, and occasionally to a generalist service (hospitalist, internal medicine, family medicine). Admitting cardiology services are staffed by both interventional trained and non-interventional cardiologists. Allocation to the admitting service was not randomized and was not performed at an individual patient level.

Patients were randomized to either invasive angiography or CMR stress imaging. Given the early enrollment, patients were typically enrolled before serial troponin values resulted, prior to determining the admitting service, and before evaluation by a cardiologist. Therefore, changes in testing modalities were allowed for safety. Interpretations of test results and decisions to perform revascularization were not directed by the study protocol. The stress CMR approach is available in the main manuscript to this study [11]. Blinding of the clinical team and participants was not performed due to the nature of the intervention. Study outcome adjudicators were blinded to the treatment arm. Data collected included demographics, cardiovascular disease risk factors, conventional troponin measurements and initial electrocardiogram (ECG) interpretation. ECGs were judged to be ischemic or not by the treating physician (patients determined to have ST-elevation myocardial infarction were excluded). These procedures are further described in the main manuscript to this study [11].

### 2.3. Outcomes

The primary outcome for this analysis was invasive angiography performed during the index-hospitalization. Secondary outcomes included adherence to protocol, rate and order of specific cardiac tests performed. Patients in the invasive-based group who received invasive angiography as the first test or second test after a resting echo were considered adherent. Patients in the CMR-based group who received CMR before invasive angiography were considered adherent. Therapeutic yield of angiography during the index visit was defined as the proportion of invasive angiography procedures that resulted in revascularization with angioplasty or coronary artery bypass grafting during the index encounter.

### 2.4. Data analysis

We describe patient demographics, risk factors and cardiac tests performed using mean and standard deviation or frequency and percentage. Stratified by admitting physician specialty, we compared age with *t*-test; sex, race, ethnicity, risk factors, and initial ischemic ECG with Fisher’s exact test; and initial troponin and peak troponin with Wilcoxon rank-sum test. Protocol adherence and use of index invasive angiography were summarized for the entire cohort, within randomization arm, and by admitting physician specialty using frequency and percentage, and were compared between interventional cardiologists and other physicians using logistic generalized estimating equations (GEEs). Odds ratios (ORs) with 95 % confidence intervals were reported for each outcome for the overall cohort and separately within each randomization arm and clustering by physician. ORs for overall adherence to protocol and overall invasive angiography were adjusted for randomization arm. Therapeutic yield was also compared between interventional cardiologists and other physicians for the overall cohort and separately within each randomization arm using a GEE approach to account for clustering by physician.

## 3. Results

Among 258 patients accrued at Wake Forest Baptist Medical Center, 40.3 % (104/258) were female, 64.7 % (167/258) were White patients, and 33.0 % (85/258) were Black with an average age of 60.7 years (SD ± 10.9). Half of patients (50.4 %; 130/258) were admitted by an interventional cardiologist. Demographics, risk factors, initial ischemic ECG, initial troponin, and peak troponin were statistically similar for patients stratified by admitting physician specialty as described in Table 1.

Invasive angiography occurred in 59.3 % (153/258) of patients during their index visit. Rates of invasive angiography were higher among patients admitted by an interventional cardiologist compared to those admitted by other physicians [65.4 % (85/130) vs. 53.1 % (68/128); aOR 1.75 (1.14–2.68), adjusted for randomization arm and clustering of individual physician]. Overall, 71.3 % (184/258) of patients received care adherent to the study protocol as randomized, with no difference seen by physician specialty. Among the 130 patients randomized to an invasive strategy, adherence to protocol was observed among 75.4 % (98/130) and was more frequent among patients admitted by interventional cardiologists [85.3 % (58/68)] than patients admitted by other physicians [64.5 % (40/62)]; aOR 2.82 (1.08–7.38). Adherence to protocol was observed among 66.7 % (86/128) of patients randomized to the CMR arm, with no significant difference seen between admitting specialties. Table 2 describes rates of protocol adherence and index invasive angiography by physician specialty and randomization arm. Table 3 describes the first and second advanced cardiac test complete by physician specialty and randomization arm.

For the entire cohort, the therapeutic yield of angiography was 43.1 % (66/153), with the therapeutic yield for interventional cardiologists and other physicians being 35.3 % (30/85) and 52.9 % (36/68), respectively, *p* = 0.31. For patients randomized to the invasive arm, the therapeutic yield overall, for interventional cardiologists, and for other physicians was 35.4 % (35/99), 27.6 % (16/58) and 46.3 % (19/41), respectively, *p* = 0.27. Among patients in the CMR arm, the therapeutic yield was 57.4 % (31/54) overall and 51.9 % (14/27) and 63.0 % (17/27) for interventional cardiologists and other physicians, respectively, *p* = 0.49.

## 4. Discussion

This is a secondary analysis of the CMR-IMPACT trial analyzing the association of physician specialty on choice of diagnostic testing modalities used to evaluate patients with detectable to mildly elevated troponin measures admitted for possible ACS. We found that patients who were cared for by an interventional cardiologist, as opposed to another physician specialty, were more likely to receive invasive coronary angiography during the admission. We observed that interventional cardiologists, compared to other admitting physicians, had a higher rate of adherence to protocol for patients who were randomized to the invasive arm. Other multifactorial reasons may additionally contribute to the difference in the rate of invasive angiography observed by physician specialty.

First, fewer barriers to using invasive angiography exist for interventional cardiologists compared to other physicians. Interventional cardiologists do not need to consult another physician to perform the procedure. This explanation is strengthened by the notably similar rate of invasive angiography between interventional cardiologists and other physicians among patients randomized to the CMR arm. Our findings therefore may be conditional on the context of the trial.

Second, there may be variation in the belief of the benefit of invasive angiography and subsequent percutaneous coronary intervention (PCI) compared to other diagnostic and treatment modalities. Previous evidence suggests that cardiologists are more likely to refer patients for catheterization than other physicians [5,6,12]. One qualitative study found cardiologists were likely to recommend PCI, even in cases when the benefits of PCI would not be superior to medical management alone [5]. In our study, one in six patients randomized to the CMR-based arm received invasive angiography as the first cardiac test, regardless of admitting physician specialty and despite guideline recommendations for intermediate risk patients suggesting anatomical or stress testing as the first testing modality [1].

Third, reimbursement potential has been observed as a potential driver for diagnostic and treatment choices even among cardiologists [8–10]. While a qualitative analysis found both interventional cardiologists and non-invasive cardiologists refuted the idea that financial gain could drive the choice to perform invasive angiography, the results of the study may have be influenced by social desirability bias [5].

Finally, in the main findings of the CMR-IMPACT study, the therapeutic yield of invasive angiography was higher among patients randomized to the CMR-based strategy compared to those randomized to the invasive angiography strategy [11]. When investigated by admitting physician subgroups in this study, the point estimate of the therapeutic yield of invasive angiography was lower among interventional cardiologists when compared to other physicians. This may be due to the less selective use of invasive angiography among invasive cardiologists. This difference was not significant; however, the study was not powered to specifically investigate this difference.

This manuscript has several limitations. Generalizability is limited by the study setting of a single, academic center and context of a randomized, controlled trial. Participants were commonly enrolled after a single troponin result. New data, such as a second troponin measurement, may have driven diagnostic choices that were nonadherent to randomization arm. The admitting service was not randomly allocated, however no predetermined clinical criteria directed the choice of admitting service. Contemporary troponin measures were used in this trial rather than high-sensitivity troponin measures. The study sample size was not designed to detect differences in practice by physician specialty, and thus may have been underpowered for this outcome, especially when stratified by randomization arm. Randomization helped direct testing choice, which may have masked real-world practices that would be observed outside of the context of an RCT.

## 5. Conclusion

Invasive angiography was more frequent among patients admitted by interventional cardiologists compared to other physicians, suggesting physician specialty may contribute to heterogeneity in cardiac test utilization.

## Figures and Tables

**Table 1:** Patient characteristics overall and by admitting specialty.

Patient characteristics	Overall n (%) or mean (SD)/median (IQR) (*N* = 258)	Interventional cardiologist n (%) or mean (SD)/ median (IQR) (*N* = 130)	Other Physician n (%) or mean (SD)/ median (IQR) (*N* = 128)	*p*-value**
Age, yrs	60.7 ± 10.9	61.7 ± 10.6	59.7 ± 11.1	0.13
Sex				
Female	104 (40.3)	50 (38.5)	54 (42.2)	0.54
Race				0.76
Native American	1 (0.4)	1 (0.8)	0 (0)	
Asian	2 (0.8)	1 (0.8)	1 (0.8)	
Pacific Islander	1 (0.4)	1 (0.8)	0 (0)	
Black or African American	85 (33.0)	47 (36.2)	38 (29.7)	
White	167 (64.7)	79 (60.8)	88 (68.8)	
Other	2 (0.8)	1 (0.8)	1 (0.8)	
Ethnicity				
Hispanic or Latino	3 (1.2)	1 (0.8)	2 (1.6)	0.62
Risk factors				
Weight, lbs	199.0 ± 43.0	195.6 ± 41.7	202.4 ± 44.2	0.20
Height, inches	67.6 ± 4.1	67.7 ± 4.1	67.6 ± 4.2	0.79
Current or history of smoking	162 (62.8)	83 (63.8)	79 (61.7)	0.72
Current or history of cocaine use	29 (11.2)	12 (9.2)	17 (13.3)	0.30
Hypertension	197 (76.4)	105 (80.8)	92 (71.9)	0.09
Diabetes	86 (33.3)	47 (36.2)	39 (30.5)	0.33
Hyperlipidemia	150 (58.1)	72 (55.4)	78 (60.9)	0.37
Prior congestive heart failure	34 (13.2)	18 (13.9)	16 (12.5)	0.75
Prior coronary artery disease	101 (39.2)	56 (43.1)	45 (35.2)	0.19
Prior MI	72 (27.9)	41 (31.5)	31 (24.2)	0.19
Prior stent/PCI	66 (25.6)	33 (25.4)	33 (25.8)	0.94
Prior CABG	35 (13.6)	20 (15.4)	15 (11.7)	0.39
Prior coronary invasive angiography	104 (40.3)	55 (42.3)	49 (38.3)	0.51
Prior cerebral vascular accident	26 (10.1)	15 (11.5)	11 (8.6)	0.43
Prior peripheral vascular disease*	20 (7.8)	12 (9.4)	8 (6.3)	0.35
Family history of ACS	113 (43.8)	55 (42.3)	58 (45.3)	0.63
ECG Interpretation*				
Ischemic ECG	27 (10.6)	17 (13.3)	10 (7.8)	0.22
Troponin				
Initial troponin, ng/ml	0.027 (0.012–0.073)	0.027 (0.011–0.095)	0.026 (0.012–0.069)	0.98
Peak troponin, ng/ml	0.044 (0.020–0.269)	0.045 (0.019–0.282)	0.042 (0.022–0.224)	0.96

SD = Standard Deviation; MI = Myocardial Infarction; PCI = Percutaneous Coronary Intervention; CABG = Coronary Artery Bypass Graft; ACS = Acute Coronary Syndrome.

* 2 patients are missing information on peripheral vascular disease and ECG interpretation.

** Continuous variables were compared between admitting specialties using *t*-tests or Wilcoxon rank-sum tests and categorical variables were compared between admitting specialties using chi-square or Fisher’s exact tests as appropriate.

**Table 2:** Protocol adherence and index invasive angiography overall, by admitting specialty, and by randomization arm.

	Overall % (n/N)	Interventional cardiologist % (n/N)	Other Physician % (n/N)	aOR* (95 %CI)	*p*-value*
Overall**					
Adherent to protocol	71.3 % (184/258)	76.9 % (100/130)	65.3 % (84/128)	1.30 (0.64–2.64)	0.46
Index invasive angiography	59.3 % (153/258)	65.4 % (85/130)	53.1 % (68/128)	1.75 (1.14–2.68)	0.01
Invasive-based					
Adherent to protocol	75.4 % (98/130)	85.3 % (58/68)	64.5 % (40/62)	2.82 (1.08–7.38)	0.03
Index invasive angiography	76.2 % (99/130)	85.3 % (58/68)	66.1 % (41/62)	2.59 (0.98–6.81)	0.054
CMR-based					
Adherent to protocol	66.7 % (86/128)	67.7 % (42/62)	66.7 % (44/66)	0.82 (0.35–1.94)	0.65
Index invasive angiography	44.4 % (54/128)	43.6 % (27/62)	40.9 % (27/66)	0.90 (0.59–1.36)	0.62

OR = Odds Ratio; CMR = Cardiac Magnetic Resonance.

* From a logistic GEE model that accounts for clustering within physician; OR compares interventional cardiologist to other physician.

** Randomization arm is included in the model.

**Table 3:** First and second advanced cardiac diagnostic testing completed by admitting physician specialty and randomization arm

Patient Characteristics	Interventional cardiologist *N* = 130		Non-interventional cardiologist *N* = 128	
	Invasive-based n (%) *N* = 68	CMR-based n (%) *N* = 62	Invasive-based n (%) *N* = 62	CMR-based n (%) *N* = 66
First cardiac test completed				
Invasive angiography	47 (69.1 %)	9 (14.5 %)	28 (45.2 %)	9 (13.6 %)
Stress echo	4 (5.9 %)	3 (4.8 %)	1 (1.6 %)	2 (3.0 %)
Resting echo	15 (22.1 %)	5 (8.1 %)	24 (38.7 %)	16 (24.2 %)
stress MRI	1 (1.5 %)	42 (67.7 %)	2 (3.2 %)	38 (57.6 %)
Stress nuclear	0 (0 %)	0 (0 %)	1 (1.6 %)	0 (0 %)
None	1 (1.5 %)	3 (4.8 %)	6 (9.7 %)	1 (1.5 %)
Second cardiac test completed				
Invasive angiography	11 (16.2 %)	18 (29.0 %)	13 (21.0 %)	14 (21.2 %)
Stress echo	1 (1.5 %)	3 (4.8 %)	1 (1.6 %)	3 (4.6 %)
Resting echo	11 (16.2 %)	4 (6.5 %)	10 (16.1 %)	8 (12.1 %)
Stress MRI	0 (0 %)	0 (0 %)	2 (3.2 %)	6 (9.1 %)
Stress nuclear	1 (1.5 %)	1 (1.6 %)	2 (3.2 %)	0 (0 %)
Stress ECG	0 (0 %)	0 (0 %)	0 (0 %)	1 (1.5 %)
None	44 (64.7 %)	36 (58.1 %)	34 (54.8 %)	34 (51.4 %)

## Appendix A. CMR-IMPACT Research Group

Subha V. Raman, MD^1^, Jeffrey M. Caterino, MD^2^, Carol L. Clark, MD^3^, Alan E. Jones, MD^4^, Michael E. Hall, MD^5^, Brian C. Hiestand, MD, MPH^6^.

^1^Ohio Health, Columbus, OH, USA; ^2^Department of Emergency Medicine, The Ohio State University, Columbus, OH, USA; ^3^Department of Emergency Medicine, Corewell Health William Beaumont University Hospital, Royal Oak, MI, USA; ^4^Department of Emergency Medicine, University of Mississippi Medical Center, Jackson, MS, USA; ^5^Department of Medicine, University of Mississippi Medical Center, Jackson, MS, USA; ^6^Department of Emergency Medicine, Wake Forest School of Medicine, Winston-Salem, NC, USA.
